# Crystallization of neomycin nanoparticles in the presence of polyvinyl pyrrolidone (PVP)

**DOI:** 10.1039/d4na01031k

**Published:** 2025-02-14

**Authors:** Sirous Motahari, Abdolmohammad Alamdari, M. Reza Malayeri

**Affiliations:** a Department of Chemical Engineering, School of Chemical and Petroleum Engineering, Shiraz University Shiraz Iran malayeri@shirazu.ac.ir

## Abstract

Neomycin nanoparticles were prepared using the inductive crystallization method in the presence of polyvinylpyrrolidone (PVP) as a stabilizer. Given the favorable solubility of neomycin in water, distilled water was used as the solvent. In addition, acetone was utilized as the antisolvent due to its high polarity and good solubility in water. The produced neomycin nanoparticles were characterized by various analyses such as TEM, HR-TEM, SEM, FE-SEM, FT-IR, XRD, DSC, TGA, AFM, DLS, and EDX. The DLS analysis indicated a bimodal size distribution from 17 to 235 nm. The induction time and nucleation mechanism were also determined. The results showed that the primary nucleation mechanism was the prevailing process, as validated by the higher *R*^2^ values. The potential role of PVP as a stabilizing agent influenced the crystallization of neomycin nanoparticles and prevented crystal aggregation, as well as favorably changing the surface tension and solubility. It was also observed that the mixing speed can affect the induction time and thus the optimal speed was set to 300 rpm. Additionally, the effect of solvent–antisolvent ratios on solubility was examined, demonstrating that higher supersaturation leads to decreased solubility of neomycin in acetone–water mixtures. Finally, the ternary diagram or two-phase nucleation related to Metastable Zone Width (MSZW) was determined.

## Abstract 2

Neomycin nanoparticles were prepared using the inductive crystallization method in the presence of polyvinylpyrrolidone (PVP) as a stabilizer.

## Graphical abstract

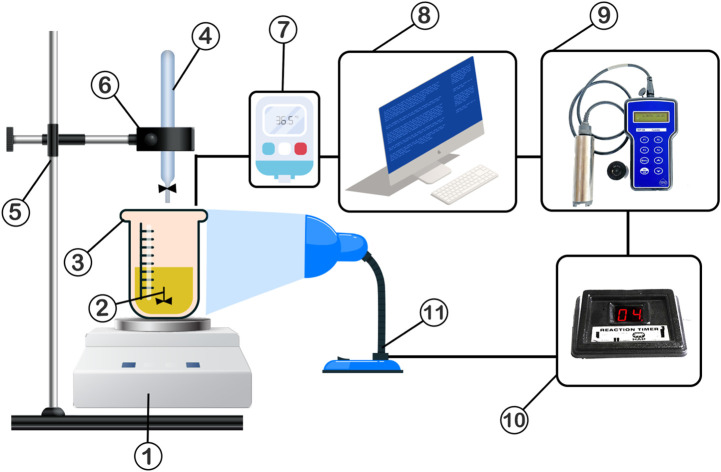

## 1. Introduction

Neomycin is an antibiotic from the aminoglycoside group that is commonly used to treat bacterial infections of the skin and intestines.^1^ It is active in the presence of Gram-negative and Gram-positive organisms. This drug stops the growth of bacteria by disrupting protein synthesis. Having said that, it may have side effects such as allergic reactions and digestive disorders, and in high doses or long-term use, it may damage the kidneys and hearing.^2,3^ It should be used under the supervision of a medical practitioner. Due to its availability, relatively low cost, and perceived efficacy, neomycin is widely used in combination with other antibiotics, antifungals, and corticosteroids.^4,5^ For this reason, its synthesis and optimizing the parameters affecting its production have always been the subject of research interest.

Nowadays, the application of nanotechnology has been widespread in various industries.^6^ Nanotechnology is not a new concept as it has now become a general purpose technology. The field of nanotechnology focuses on sub-molecular structures.^7^ These nanomaterials with dimensions between 0.1 and 100 nanometers exhibit unique mechanical, electrical, magnetic, thermal and imaging properties which have led to their use by researchers in various medical and pharmaceutical fields of research and applications.^8–10^ In the pharmaceutical industry, manufacturers need to produce high-quality products (including size, purity, and morphology), with long-term stability and repeatable properties.^11^ Therefore, drug nanocrystals especially for poorly water-soluble drugs have been considered due to their size reduction benefits such as improved solubility and bioavailability, increased dissolution rate, and further purification.^12–14^ Nanoparticles have also been developed recently to create moderately soluble compounds to enhance their properties in cosmetic products such as dermal penetration.^15–17^

Different methods are commonly used to synthesize nanoparticles, including the sono-chemical process, the sol–gel method, the reaction of solid–liquid states, condensation of the gas phase, and chemical precipitation.^18–20^ Among these methods, chemical precipitation from the liquid phase is one of the simplest and most common methods to obtain nanoparticles due to advantages such as the formation of pure and homogeneous materials.^21–23^ The general principles of chemical precipitation reactions are based on the process of converting soluble species in the solution to insoluble species in the presence of precursors.^24^ In this method, two parameters are usually considered: the mechanism of nucleation and the induction time.^25^ To calculate nucleation, both induction time and supersaturation must be measured.^26^ In the crystallization process, the time between supersaturation and the first changes in the physical properties of the system is defined as the induction time. This is due to the appearance of new particles and detectable changes in the mixture. These changes included viscosity, conductivity, and color of the solution.^23,27^ Induction time can be used to estimate the nucleation rate and amplitude of the metastable zone width in the crystallizer.^28,29^

Antisolvent precipitation is a well-known method for producing nanoparticles due to its simple crystallization process, operation at ambient temperature, and low energy consumption.^30–32^ This method consists of two stages: phase separation, where crystals are formed, and crystal growth. In antisolvent or inductive crystallization, supersaturation is achieved by introducing an antisolvent into the system. The presence of an antisolvent can have two important impacts: firstly, it reduces the solubility of a solute in the solution, which, in turn, facilitates the rapid crystallization process. Secondly, the antisolvent quickly induces a high level of supersaturation, which results in accelerated nucleation rates. This increased nucleation rate leads to the formation of small particles.^33,34^

Understanding the mechanism of nucleation will help manufacturers to increase the chances of producing the desired product using a suitable crystallizer.^11,35,36^ There are various techniques to discern the nucleation kinetics and related parameters used in previous research, and the appropriate method should be carefully considered.^30^ The induction time is influenced by parameters such as supersaturation and temperature.^37^ The morphology of nanoparticles depends on certain conditions such as nucleation and growth rate.^38,39^ Therefore, the type of additive can affect the quality of synthesized nanoparticles. In what follows, the application of various additives in the synthesis of nanoparticles using the crystallization method has been investigated.

Wang *et al.*^40^ investigated the inhibiting effect of three different types of polymers including polyvinylpyrrolidone (PVP), Kollidone and hydroxyl propyl methyl cellulose (HPMC) on the crystal growth and nucleation of indomethacin from various supersaturated solutions. They reported that PVP has a relatively stronger effect on inhibiting crystal growth at low concentrations and the inhibition of crystal growth by polymers may be due to a delay in drug surface integration. The specific impact may vary depending on the polymer, and the inhibition of both nucleation and crystal growth by polymers should be considered when studying drug inhibitors.^41^ The synthesis of Cu–Au alloy nanoparticles using antisolvent crystallization in the presence of PVP as a stabilizer has been investigated by Liu *et al.*^42^ They used a new two-step method based on antisolvent crystallization for the synthesis of nanoparticles; the first step included the synthesis of Cu and Au precursors in ethanol, and in the second stage the crystallization process has been carried out in the presence of PVP as a stabilizer. Their results showed that in the used method, the synthesized nanoparticles were not affected by the experimental conditions (concentration and temperature during the reaction).

Haghighizadeh *et al.*^43^ have reviewed the recent developments in the field of nanoparticle synthesis using the antisolvent crystallization process. Antisolvent crystallization of papain was investigated by Boonkerd *et al.*^44^ They used organic solvents such as ethanol, acetone and acetonitrile as antisolvents and investigated the effect of parameters such as solvent to antisolvent volume ratio and papain concentration. They reported that ethanol antisolvent performed better than other used antisolvents and its optimal performance conditions were obtained at a concentration of 30 mg ml^−1^ of papain and a volume ratio of 1 : 4 (solvent to antisolvent). Under the mentioned conditions, papain crystals were spherical with an average size of 207.6 nm and a crystallization yield of approximately 80%. Antisolvent crystallization of poorly water soluble drugs has been studied by Abhijit *et al.*^45^ The recrystallization of caffeine particles during the antisolvent crystallization process has been done by Torkian *et al.*^27^ They used chloroform (CHCl_3_) and carbon tetrachloride (CCl_4_) as solvent and antisolvent, respectively, and have investigated the effect of operating parameters on induction time such as temperature, feeding speed, stirring speed and stabilizer concentration. The results showed that under high supersaturation conditions, the dependence of induction time on supersaturation is less, which can be due to the importance of growth ability and secondary nucleation time.

In this study, neomycin nanoparticles have been synthesized through the induced crystallization process in the presence of PVP as a stabilizer. The supersaturation is achieved by adding a controlled amount of the antisolvent to the solution. The synthesized nanoparticles were then characterized using various analytical techniques such as Scanning Electron Microscopy (SEM), Field-Emission Scanning Electron Microscopy (FESEM), Transmission Electron Microscopy (TEM), High Resolution Transmission Electron Microscopy (HR-TEM), Fourier Transform Infrared Spectroscopy (FTIR), X-ray Diffraction (XRD), Atomic Force Microscopy (AFM), Dynamic Light Scattering (DLS), Thermo-Gravimetric Analysis (TGA), Differential Scanning Calorimetry (DSC), and Energy Dispersive X-ray (EDX or EDS) spectroscopy. The induction time has experimentally been measured using *in situ* turbidimeter and reaction timer (visual) methods and their results were compared. Then, the solubility of neomycin, the nucleation mechanism and the effect of supersaturation and agitation rate on the induction time have been investigated. In order to investigate the effect of the initial concentration of PVP solution on the nucleation mechanism, the interfacial energy has been calculated for two different concentrations of PVP. Finally, the ternary diagram or two-phase nucleation related to the Metastable Zone Width (MSZW) was creatively plotted using MATLAB software.

## 2. Theory

### 2.1. Classical nucleation model

Classical nucleation is a theoretical framework that describes how new phases (like solid, liquid, or gas) form within a parent phase during phase transitions. It provides a model for understanding the process of nucleation, particularly how small clusters (nuclei) of a new phase emerge and grow under certain conditions. In this theory, the steady-state nucleation rate, which represents the total number of nuclei formed per unit time and volume, is described by the following Arrhenius equation:1
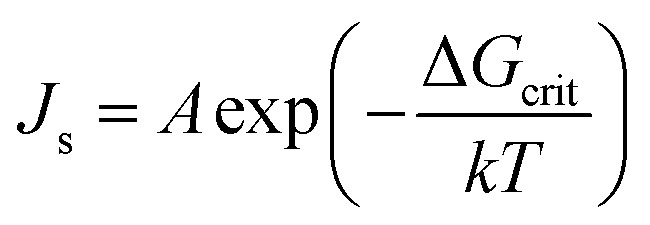
where *A* is the pre-exponential coefficient that depends on the kinetics of nucleation in the growth medium, and *k* and *T* are Boltzmann's constant and temperature (K), respectively. It should be noted that the relationship between temperature and the supersaturation ratio (*S*) is presented in eqn (2).2
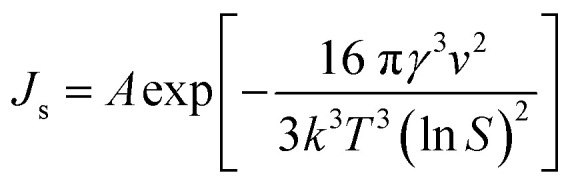


Considering the inverse relationship between the induction time and nucleation rate,^28,46^ the following equation can be derived to describe homogeneous primary nucleation. Here, the induction time refers to the time that the system needs before starting a stable nucleation process.3
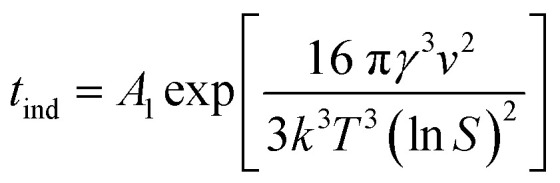


In this equation, *A*_1_ is the nucleation constant, *S* is the supersaturation ratio, *T* is the temperature, *k* is the Boltzmann constant, *γ* is the solid–liquid surface tension and _*ν*_ is the molecular volume.

The proposed model for secondary nucleation in the absence and in the presence of a solid phase in the system is presented with the following classical power equation:^47^

4 *J* = *K*_s_*S*^*n*^

in which *K*_s_ is the experimental constant of the secondary nucleation rate and *n* is the degrees of secondary nucleation.^48,49^ The highest value for *n* has been stated by Garside *et al.*^48^ to be 3.0.

In crystallizers that do not employ seeding, nuclei can form from the solute found in the mother phase. This process occurs especially in the boundary region close to the growing crystal, where there is a high concentration of solutes and ideal conditions for primary nucleation. For secondary nucleation in the crystallizer without seeds, the induction time can be presented in the following form:

5 ln *t*_ind_ = ln *K*_s_ − *n* ln *S*

In this equation, by plotting ln *t*_ind_*vs.* ln *S* and finding the slope of the line, the degree of secondary nucleation (*n*) can be estimated.

### 2.2. Interfacial energy calculations

To calculate interfacial energy, classical nucleation theory can be modified for the solid–liquid system in the following form:^29^6
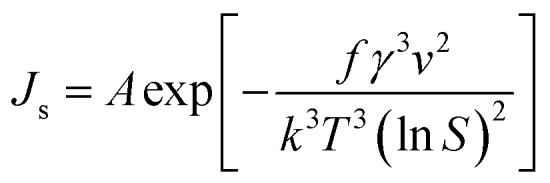
in which *f* is the particle shape factor, *γ* is the interfacial energy of the solid–liquid phase (J m^−2^) and other parameters are the same as in eqn (3). According to the inverse relationship between the nucleation rate and induction time, eqn (6) can be rewritten as follows:7
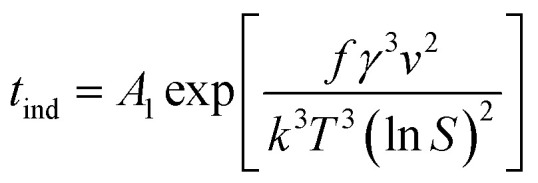


If one takes the natural logarithm from eqn (7), then8
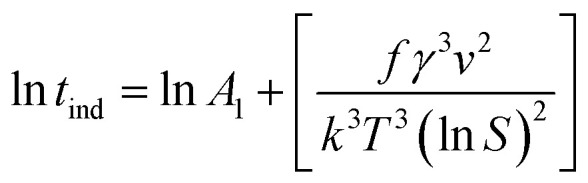


Therefore, by plotting ln *t*_ind_ against 1/(*T*^3^(ln *S*)^2^) and finding the slope of the line (*A*), interfacial energy can be calculated as:9
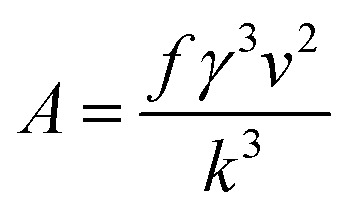


Therefore, the interfacial energy can be given by:10
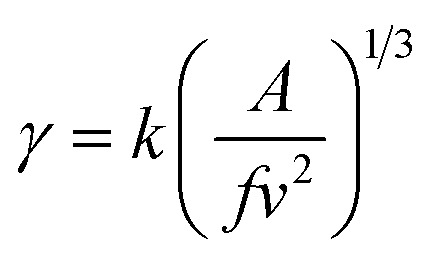
in which 
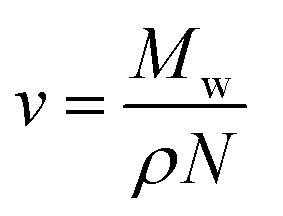
 is the molecular volume (m^3^ per molecule). Furthermore, *ρ*, *M*_W,_*k* and *N* are the density (kg m^−3^), molecular weight (kg mol^−1^), Boltzmann constant (1.38 × 10^−23^ m^2^ kg (s^2^ k)^−1^) and Avogadro number (6.022 × 10^23^ mol^−1^), respectively.

## 3. Materials and experimental procedure

### 3.1. Materials

Neomycin sulfate was purchased from Sigma-Aldrich. Polyvinylpyrrolidone (PVP) and acetone (CH_3_COCH_3_) were provided by Merck.

### 3.2. Material characterization

The surface morphology of the nanoparticles was characterized using a FE-SEM and SEM equipped with an EDX analyzer. The crystallinity was investigated by XRD. FT-IR analysis as well as TEM and HRTEM analyses were performed.

### 3.3. Preparation of PVP solution

In order to prepare the PVP solution, 1.0 g of PVP powder (*M*_W_ = 111.14 g mol^−1^) was dissolved in 1.0 liter of double-distilled water and stirred well to prepare an aqueous solution with a concentration of 8.99 mM. This solution was kept at room temperature for two days and stirred twice daily to obtain a uniform and stable solution of PVP. The final PVP solution was then used as a stabilizer in the synthesis of neomycin nanoparticles.

### 3.4. Synthesis of neomycin nanoparticles

In this section, the method for the synthesis of neomycin nanoparticles is explained in detail. First, a specific amount of neomycin had to be weighed using a digital balance (accuracy 0.1 g) based on its equilibrium concentration. Then the weighed neomycin was added to 10 g of double distilled water in a beaker and mixed with a magnetic stirrer for 20 minutes at a speed of 300 rpm to obtain a homogeneous solution.

**Fig. 1:**
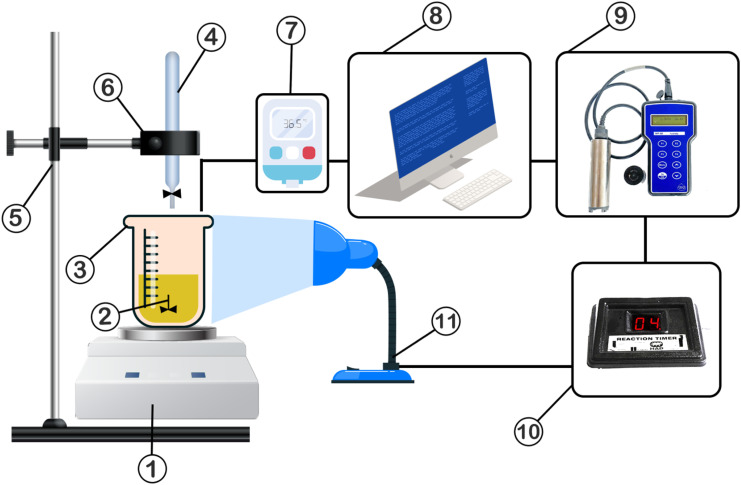
Schematic diagram of the used laboratory setup ((1) stirrer, (2) mixer or (magnetic stirrer), (3) crystallizer, (4) burt, (5) base, (6) laboratory clamp, (7) thermometer, (8) Pc lab, (9) *in situ* turbidimeter, (10) reaction timer and (11) LED).

After ensuring the homogeneity of the neomycin solution, a certain amount of the PVP solution prepared in the previous step was added to the crystallizer system and stirred continuously. To accurately control the temperature of the solution, a digital thermometer was connected to the crystallizer and a turbidimeter sensor was also set to measure the turbidity of the solution during the experiment. In the next step, acetone was slowly added to the neomycin–PVP solution in the crystallizer every 3 minutes as an antisolvent. The first physical changes (saturation stage) were observed by adding the first droplet of acetone. The solution continued to be stirred until complete turbidity (supersaturation) was obtained. The time between the first physical changes and reaching full turbidity was recorded with two methods: *in situ* turbidimeter and reaction timer, and this interval is known as induction time. The schematic diagram of the used laboratory setup is shown in Fig. 1. All experiments were repeated three times at a constant temperature of 25 °C to ensure the reproducibility and accuracy of the results.

It is worth mentioning that in this study, water was used as solvent and acetone as antisolvent. Supersaturation was achieved by introducing the antisolvent (acetone) into the system. The solute (neomycin) is insoluble in the antisolvent (acetone), while the antisolvent was totally miscible with the primary solvent (water). When acetone was added, the solubility of neomycin decreased due to the distinct polarities of water and acetone.^33^

### 3.5. Measurement of induction time

Induction time is a key parameter that is influenced by supersaturation, temperature and stirring speed.^27^ As stated earlier, this time is actually the interval between the occurrence of visible physical changes and the onset of supersaturation in the system, which is associated with the formation of crystals. By measuring the induction time, it is possible to estimate the nucleation rate, the amplitude of the MSZW and the surface tension in the crystallizer.^50^ In order to compare the two methods of measuring the induction time, reaction timer (visual) and *in situ* turbidimeter, experiments were performed under similar supersaturation conditions. The results showed that the average induction time recorded with an *in situ* turbidimeter was less than the time recorded with the reaction timer. The reason for this difference is mainly due to human errors and delays in the reaction timer. Furthermore, the standard deviation of the turbidimeter method was lower, which indicates the greater accuracy of this method. For this reason, turbidimeters are mostly used in laboratories to measure induction time.^51^ The results of the mentioned experiment are presented in Table 1.

**Table 1:** Comparison between the induction time measurement using two methods: visual method and *in situ* turbidimeter

Supersaturation (*S*)	Number of repeats	Induction time (s) (visual method)	Standard deviation	Induction time (s) (turbidimeter method)	Standard deviation
1.31	7	94	9	78	6
1.33	7	83	7	67	5
1.358	7	84	8	59	4
1.38	7	76	7	50	4
1.43	7	71	6	47	4
1.48	7	59	6	39	4
1.51	7	53	7	36	3
1.66	7	49	5	31	3
1.67	7	44	5	27	2
1.76	7	37	4	23	2

The induction time, a critical parameter in crystallization, consists of three main components: relaxation time (time required for the system to reach the quasi-stable region), nucleation time (time required for nuclei to form), and growth time (time required for nuclei to grow to a detectable level). After the addition of acetone droplets, physical changes in the solution, such as increased turbidity, were observed with a slight delay, which represented the induction time. During this stage, at lower supersaturation levels, molecular clusters reached the stability boundary and transitioned to the quasi-stable region. In this region, nuclei form through aggregation, and their growth became detectable due to increased collision energy, crystal–solution interactions, or crystal–crystal and crystal–stirring collisions, ultimately leading to secondary nucleation.

**Fig. 2:**
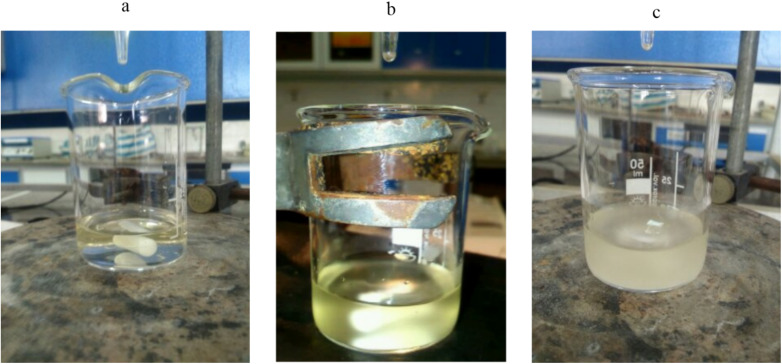
The steps of crystallization: (a) before adding the antisolvent; (b) antisolvent and saturation drop moment; (c) supersaturation.

By introducing acetone (antisolvent) into the system, the dissolved neomycin exceeded its solubility limit, creating a supersaturated state. Supersaturation, as the initial step of crystal formation, plays a crucial role in inducing primary nucleation, which can be either homogeneous or heterogeneous. With continued addition of acetone, higher supersaturation occurred, causing the formation of critical clusters and critical nuclei (see Fig. 2).^52,53^

The induction time is influenced by factors such as agitation rate, supersaturation level, impurities, and temperature. The optimization of stirring speed and the addition rate of acetone is therefore essential for achieving the desired induction time and supersaturation levels.

The crystallization process is governed by transitions through three regions—stable, metastable, and unstable—depicted in solubility diagrams and two-phase nucleation (MSZW) graphs. In the stable region, the initial addition of acetone does not induce significant changes due to minimal molecular interactions. However, with continued addition of acetone, the system would be transferred to the metastable region, where molecular interactions increase, and nuclei begin to form at the boundary of the stable and metastable regions.

As acetone concentration increased further, the stability and aggregation of neomycin molecules in the metastable region increased, leading to the formation of clusters and critical nuclei. In this region, moderate supersaturation is gradually consumed by growing nuclei. Upon reaching the boundary of the unstable region, higher levels of supersaturation are achieved, exceeding the solubility limit. This elevated supersaturation results in higher nucleation rates, which in turn reduced crystal growth and the formation of a larger quantity of smaller-sized crystals.^33,54^

## 4. Results and discussion

### 4.1. Characterization of neomycin nanoparticles

**Fig. 3:**
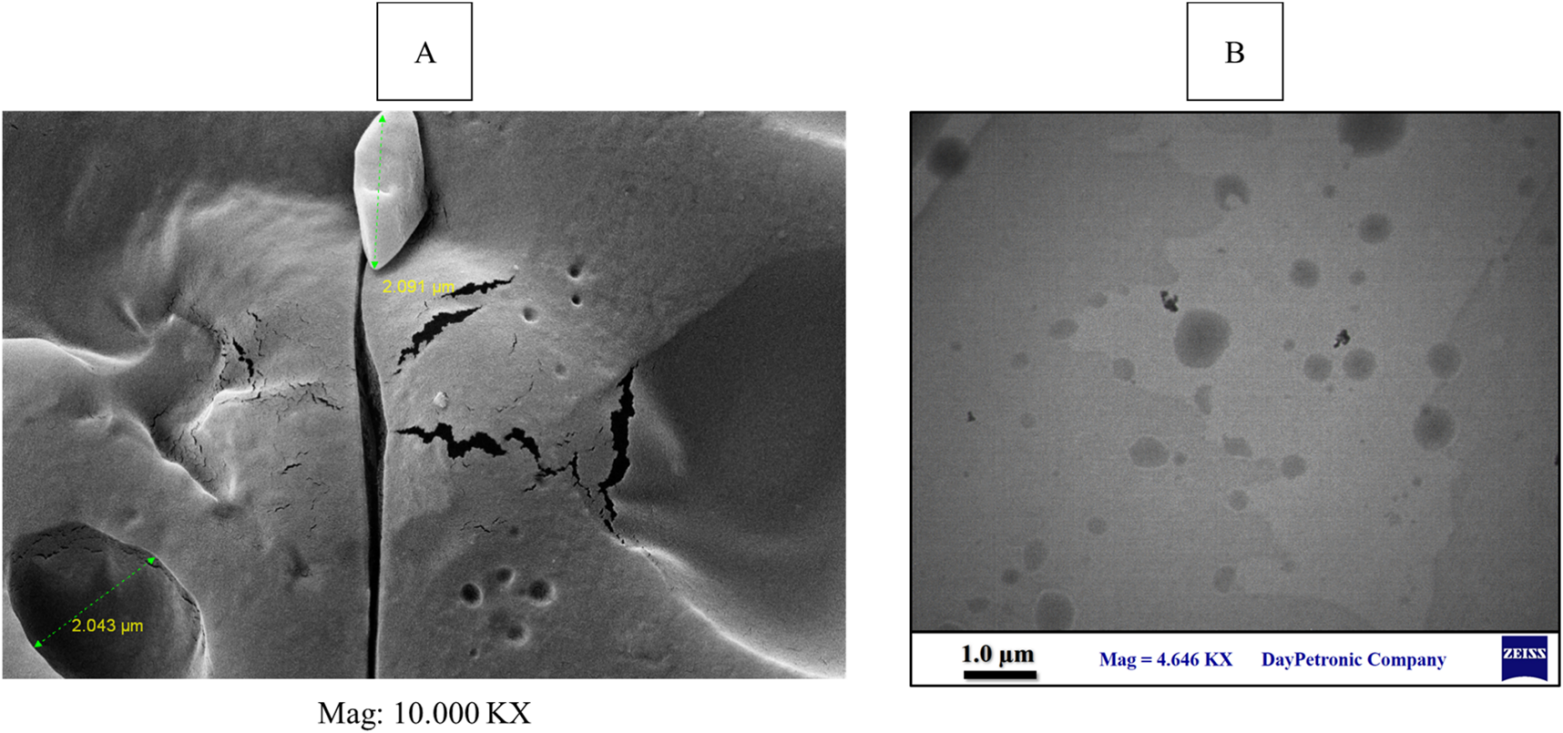
(A) SEM and (B) TEM images of nanoparticles without PVP as the stabilizer.

Initially, the synthesis of nanoparticles was carried out without the use of a stabilizer. After analyzing the SEM and TEM results, the morphology and images were found to be unsuitable due to the agglomeration of nanoparticles; therefore, all subsequent results were presented in the presence of PVP as the stabilizer. These results can be attributed to DLVO theory, which states that forces between particles in a solution are a combination of van der Waals attraction and electrostatic repulsion. When van der Waals attraction is stronger than electrostatic repulsion, particles tend to agglomerate. This behavior was evident in neomycin nanoparticles without a stabilizer due to their high surface-to-volume ratio.^55^ To address this issue, PVP was used as the stabilizer. The large neomycin molecules adhere to the particle surfaces, and when the particles come close to each other, the entanglement of PVP macromolecules prevents them from sticking together and forming aggregates. TEM and SEM images of nanoparticles without the stabilizer (PVP) are presented in Fig. 3.

**Fig. 4:**
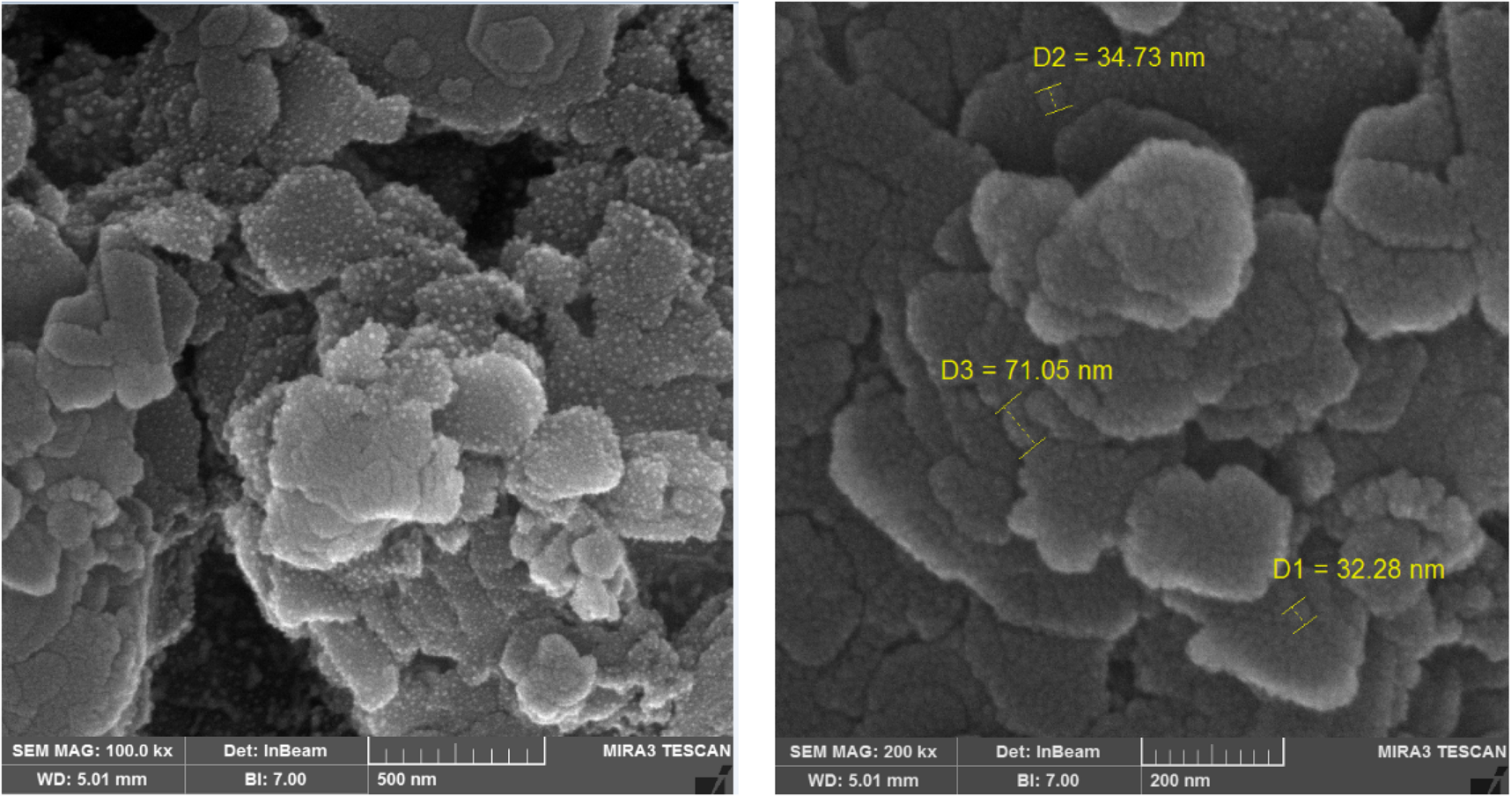
SEM imaging of the synthesized neomycin nanoparticles.

**Fig. 5:**
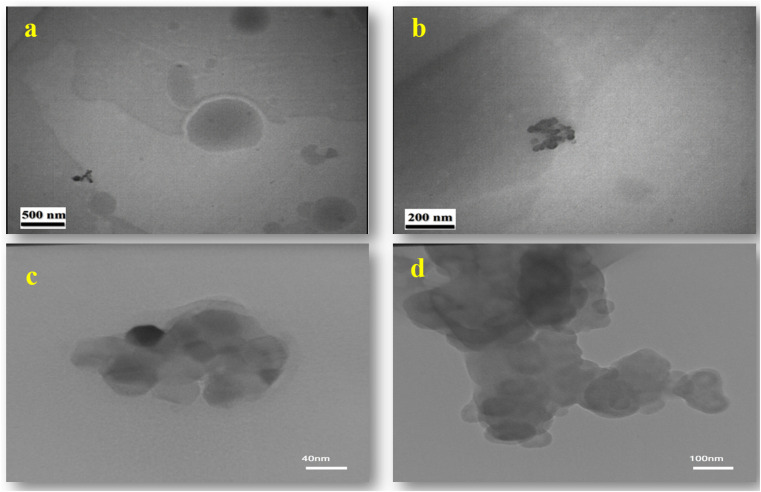
TEM at (a) 10 K and (b) 27.8 K and HR-TEM at (c) 600 K and (d) 230 K of the synthesized neomycin nanoparticles.

**Fig. 6:**
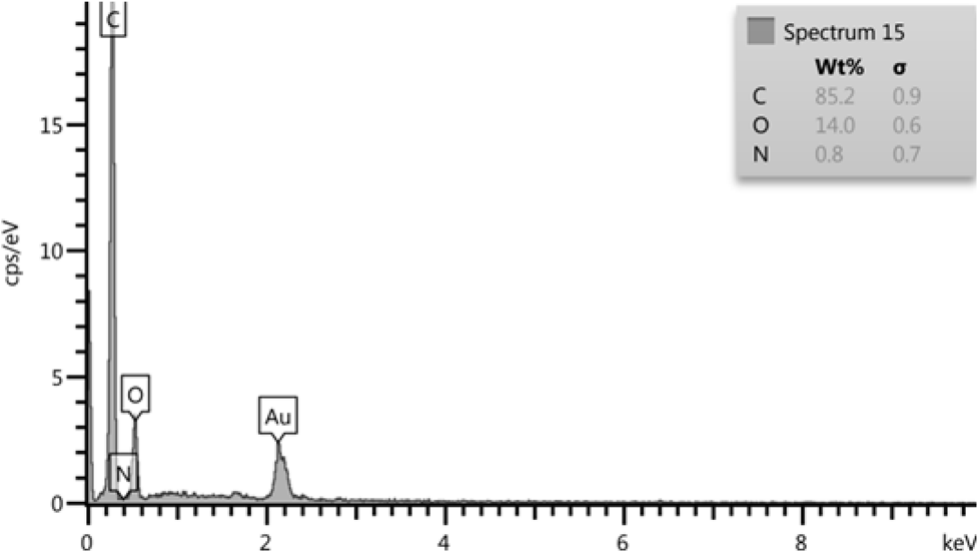
EDX spectrum of neomycin nanoparticles.

To confirm and identify the synthesized nanoparticles (with the PVP stabilizer), SEM, FESEM, TEM, HR-TEM, FTIR, XRD, AFM, and EDX analyses were conducted. The morphologies of the nanoparticles were examined using SEM, TEM, and HR-TEM imaging techniques and the collected images are reported in Fig. 4 and 5. As can evidently be seen, proportional, desirable, and well-formed particle sizes with ideal morphology of nanoparticles with optimal distribution in the presence of PVP stabilizer solution have been formed. The elemental composition was determined through EDX analysis (see Fig. 6) which confirmed the presence of C, O, N, and Au elements. The Au element detected in the spectrum was from the gold coating used in sample preparation, while the others were indicative of homogeneous distribution of the synthesized neomycin nanoparticles in the presence of surface active agents (PVP).

**Fig. 7:**
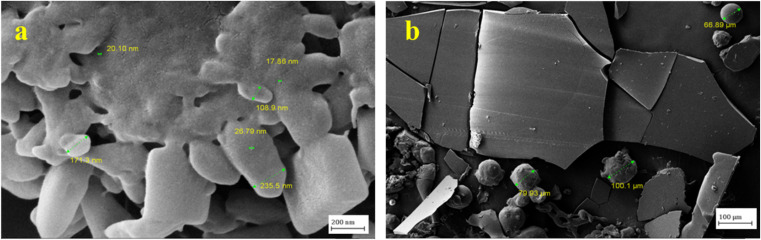
FESEM images of the synthesized neomycin nanoparticles after being lyophilized with magnifications of (a) 50k and (b) 10k times.

Moreover, the nanoparticles were lyophilized, and their FESEM images were obtained (Fig. 7) and showed the particle shape and uniform distribution.

**Fig. 8:**
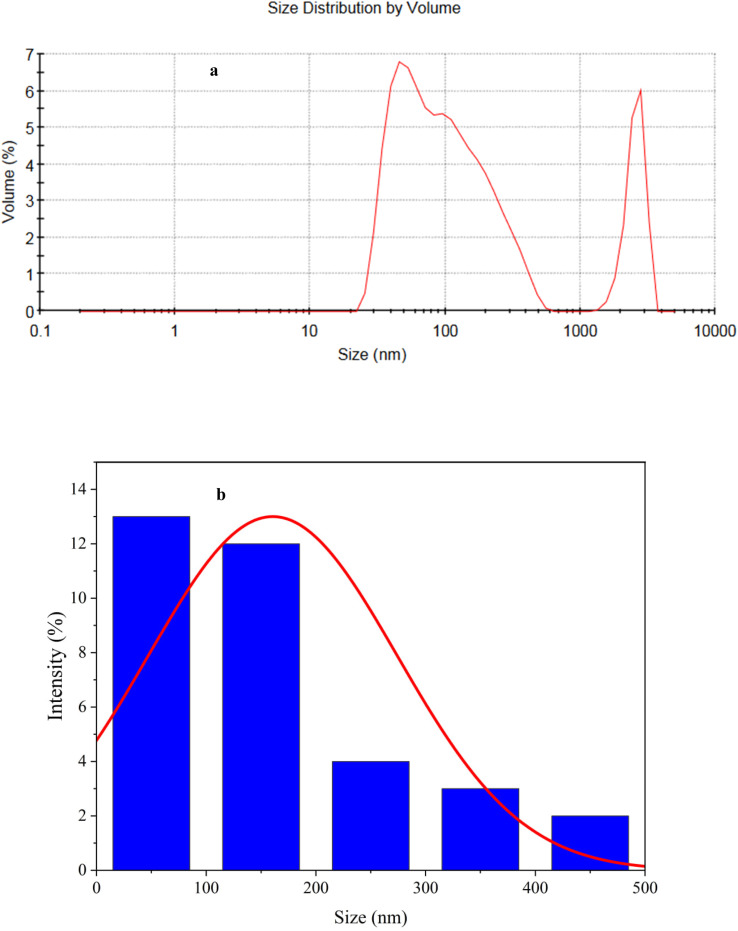
DLS diagram (a) and size distribution (b) for the synthesized neomycin nanoparticles.

The lyophilized nanoparticles were also analyzed using DLS to obtain information about the size distribution of nanoparticles (Fig. 8). According to the DLS analysis, the nanoparticles have a bimodal size distribution (Fig. 8a). The bimodal size distribution observed in this study may be attributed to the competing effects of antisolvent addition (acetone) on supersaturation and the dynamic nature of crystal growth. This suggests that the addition of acetone as an antisolvent affects the supersaturation of the system, leading to the formation of two distinct size distributions of crystals.^56^

**Fig. 9:**
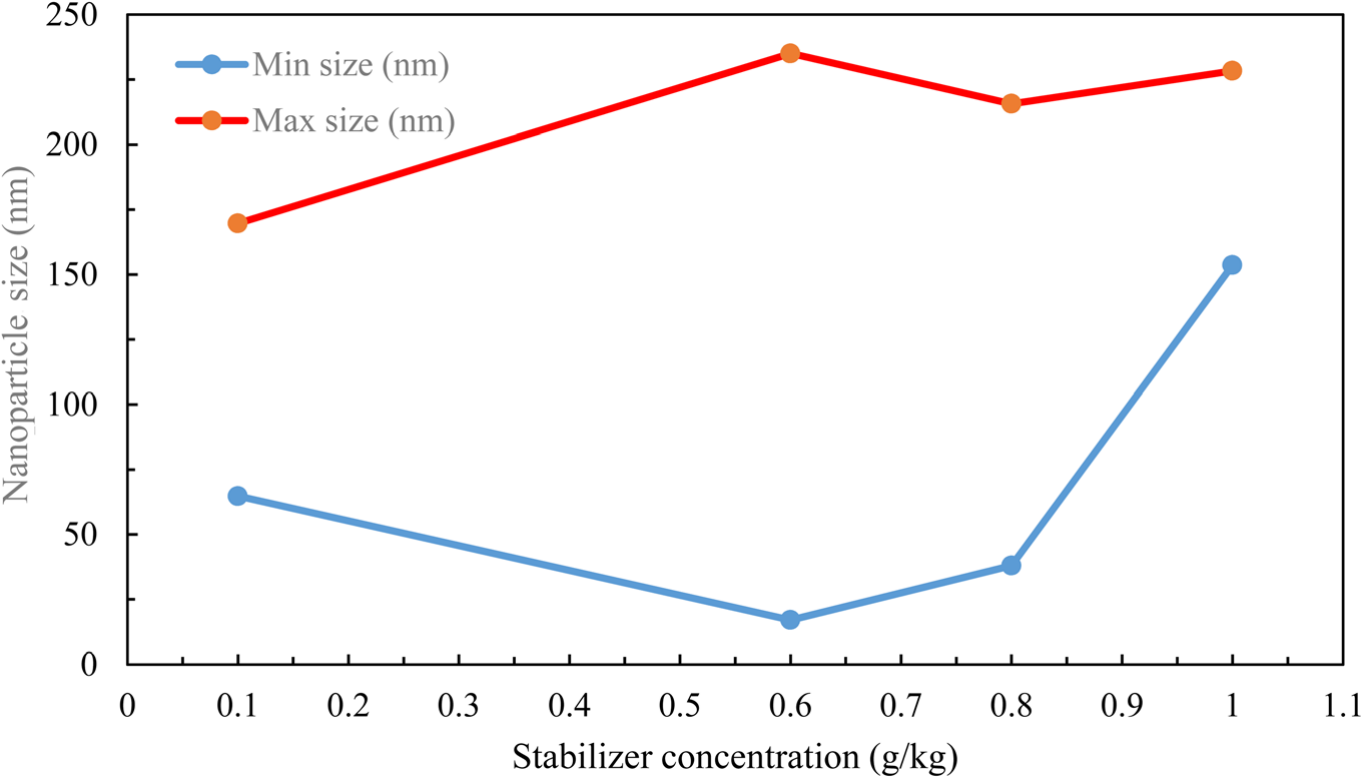
Nanoparticle size *vs.* stabilizer concentration.

As shown in Fig. 8b, the mean nanoparticle size for neomycin nanoparticles was 107.5 nm with a considerable size distribution ranging from 17 to 235 nm. This range of particle sizes was corroborated by DLS, SEM, FESEM, TEM, and HR-TEM, thus indicating the successful formation of neomycin nanoparticles. It is worth mentioning that four different concentrations (0.1, 0.6, 0.8, and 1 g kg^−1^) were examined to determine the optimal PVP concentration. Nanoparticle size ranges were: 64.76–169.7 nm (0.1 g kg^−1^), 17–235 nm (0.6 g kg^−1^), 37.96–215.6 nm (0.8 g kg^−1^), and 153.6–228.3 nm (1 g kg^−1^). Considering size, morphology, and stability, the optimal PVP concentrations were determined to be 0.6 and 0.8 g kg^−1^. The findings are illustrated in Fig. 9.

**Fig. 10:**
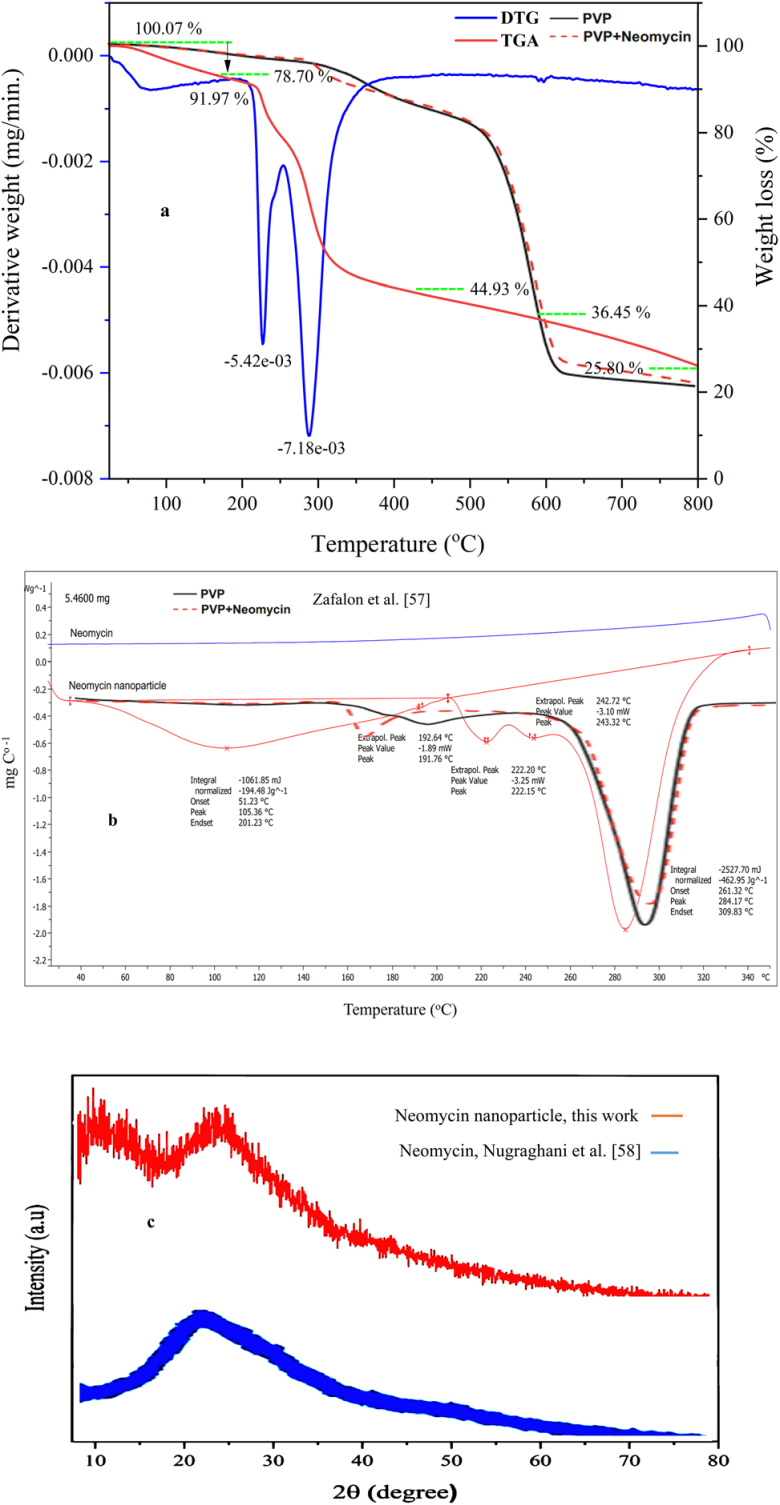
TGA (a), DSC (b) and XRD (c) spectra for the synthesized neomycin nanoparticles and comparison with existing results.^59^

To identify the thermal properties of the nanoparticles, TGA and DSC analyses were provided and the resulting curves are presented in Fig. 10a and b. The TGA curve shows that the weight loss of the nanoparticles at 190 °C is equal to 8.03%, which can be attributed to their water loss. The subsequent weight loss peaks at 227 °C and 288 °C indicate structural degradation of the nanoparticles. It can therefore be inferred that the neomycin nanostructure remains stable up to a temperature of about 230 °C as reported by other studies.^57,58^ In the DSC curve, a wide peak observed in the range of 105 °C corresponds to the melting of neomycin. The peaks seen at 222 °C and 284 °C, as shown in the TGA curve, indicate material degradation.

The X-ray diffraction (XRD) pattern of neomycin nanoparticles reveals five distinct peaks at 2*θ* = 6.67°, 7.87°, 11.09°, 19.5°, and 20.30° (Fig. 10c). These peaks match standard or characteristic peaks reported in the literature, indicating the formation of a novel crystalline phase.^59^ The differences in peak positions could be attributed to the presence of PVP, which can alter the crystal lattice and influence the growth kinetics during the crystallization process.^60,61^ Moreover, the changes in peak intensities may also reflect variations in the crystallinity and defects within the crystal structure.^62^

**Fig. 11:**
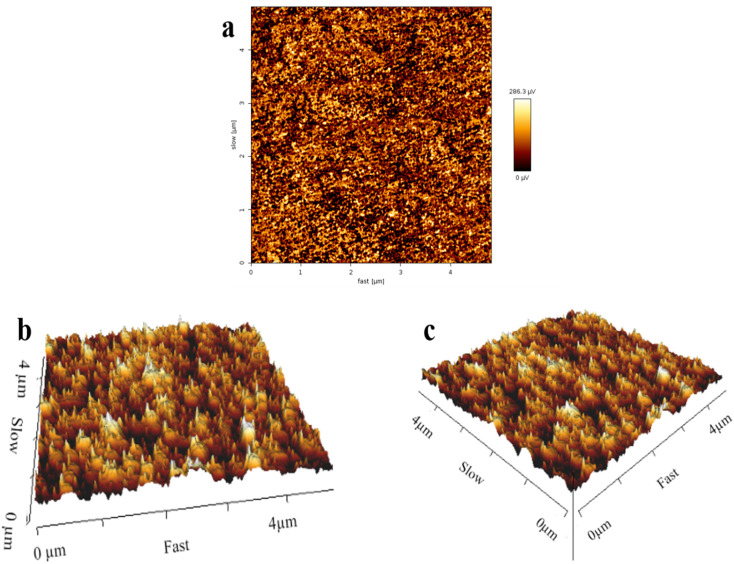
(a–c) AFM images of the neomycin nanoparticles.

To investigate the surface roughness of the synthesized nanoparticles, AFM analysis was performed and the results are presented in Fig. 11(a–c). Three-dimensional atomic force microscopy (3D AFM) has emerged as a powerful technique for characterizing the surface structure and roughness of neomycin crystals. AFM enables the study of growth mechanisms and kinetics through time-dependent imaging, offering valuable insights into the crystallization process.^63^ Both two-dimensional and three-dimensional AFM imaging facilitate the identification of crystal surfaces, roughness, defect structure, and defect density.^63^ Moreover, 3D AFM allows for the acquisition of high-resolution images of crystal surfaces in the *x*, *y*, and *z* directions, providing detailed information about the size, shape, and orientation of surface features.^64^ The AFM results underline that the neomycin nanoparticles exhibit better quality in the presence of the stabilizer, showing uniform surface roughness of the synthesized nanoparticles. The unevenness of the surface of the neomycin nanoparticles is consistent with the reported results in the literature.^65^

**Fig. 12:**
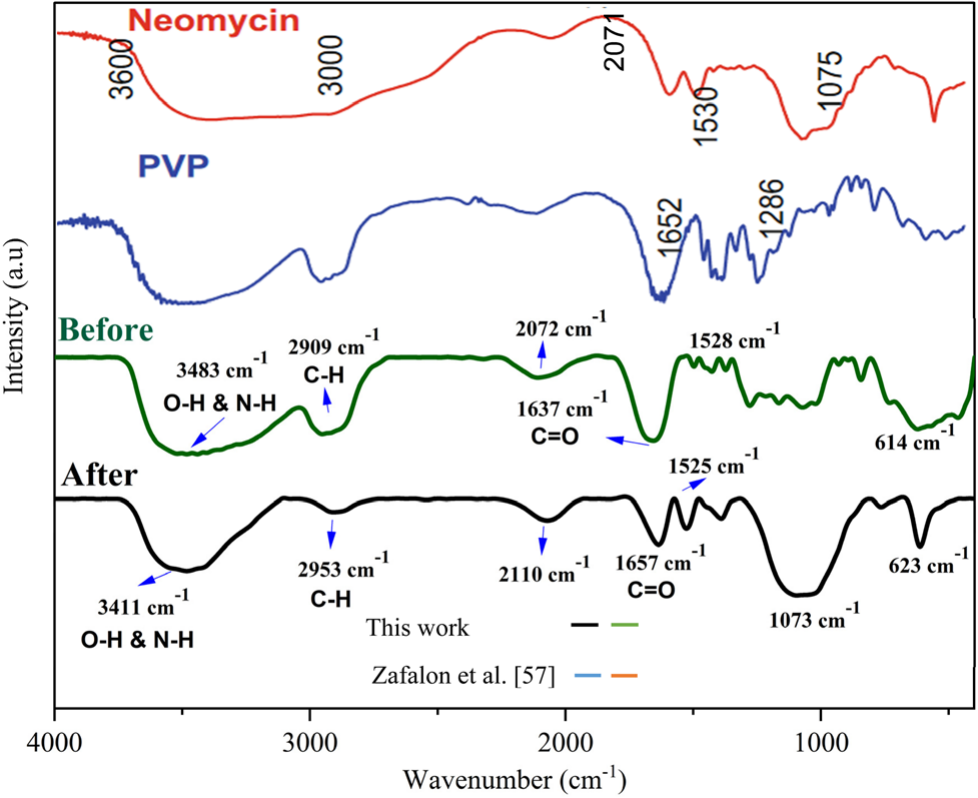
FT-IR spectroscopy before and after neomycin nanoparticle formation and comparison with existing results.^58^

FT-IR spectroscopy was considered another technique to gather additional information about the synthesized neomycin nanoparticles. In this regard, FT-IR spectra in two different steps, before the formation of neomycin nanoparticles and after the formation of neomycin nanoparticles in the presence of the PVP surfactant. were recorded (Fig. 12). The significant peaks are presented in Fig. 12 and confirm the N–H, C–H, C=O and O–H bonds in the neomycin structure, consistent with previously reported results,^59,66,67^ in which polymeric hydrogel based wound healing systems were prepared using the neomycin drug followed by gamma irradiation to promote crosslinking and sterilization.

### 4.2. Effect of agitation rate on induction time

The agitation rate of the solution is a major parameter that can affect the quality and synthesis time in the nanoparticle crystallization process.^68^ Increasing the speed of the stirrer will lead to the reduction of the induction time due to the improvement of the diffusion coefficient and the reduction of the mass transfer resistance.^69^ In this regard, a series of experiments were conducted to investigate the effect of stirring speed on the induction time of neomycin nanoparticles at a constant temperature of 25 °C and a supersaturation ratio (*S*) of 1.33. Five different stirring speeds (300, 350, 400, 450 and 500 rpm) were used and each experiment was repeated three times to obtain reliable results.

**Fig. 13:**
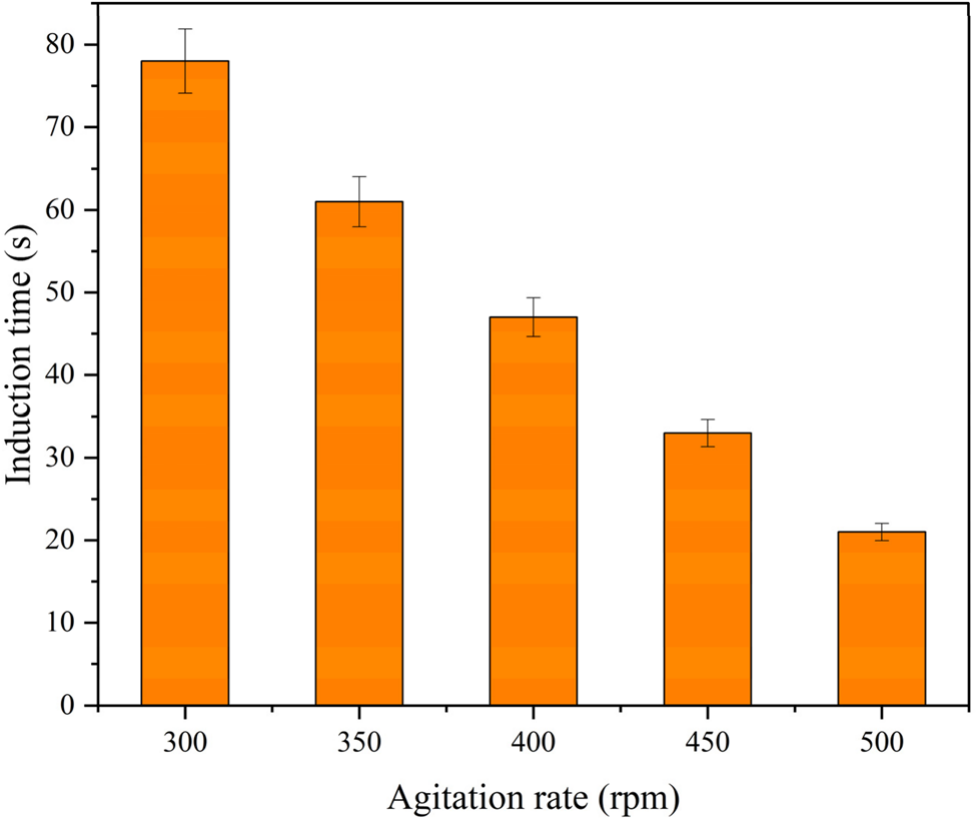
Measured induction time for five different stirrer speeds of 300, 350, 400, 450 and 500 rpm.

The results showed that the induction time decreased profoundly with increased stirring speed, as shown in Fig. 13. Increased stirring speed creates more turbulence in the system and increases the diffusion coefficient, which can reduce the mixing time and supersaturation. As a result, the mass transfer at the interfaces would noticeably be reduced and the penetration of PVP stabilizing molecules into the neomycin crystals is delayed. This situation makes the optimal supersaturation ratio to be maintained and the induction time to be shortened.

Based on the obtained results, the optimal stirring speed of 300 rpm was selected for all experiments. It should be noted that at the desired speed, sufficient stabilizer penetration and effective control of supersaturation, nucleation and induction time can be ensured.

### 4.3. Measurement of neomycin solubility

**Fig. 14:**
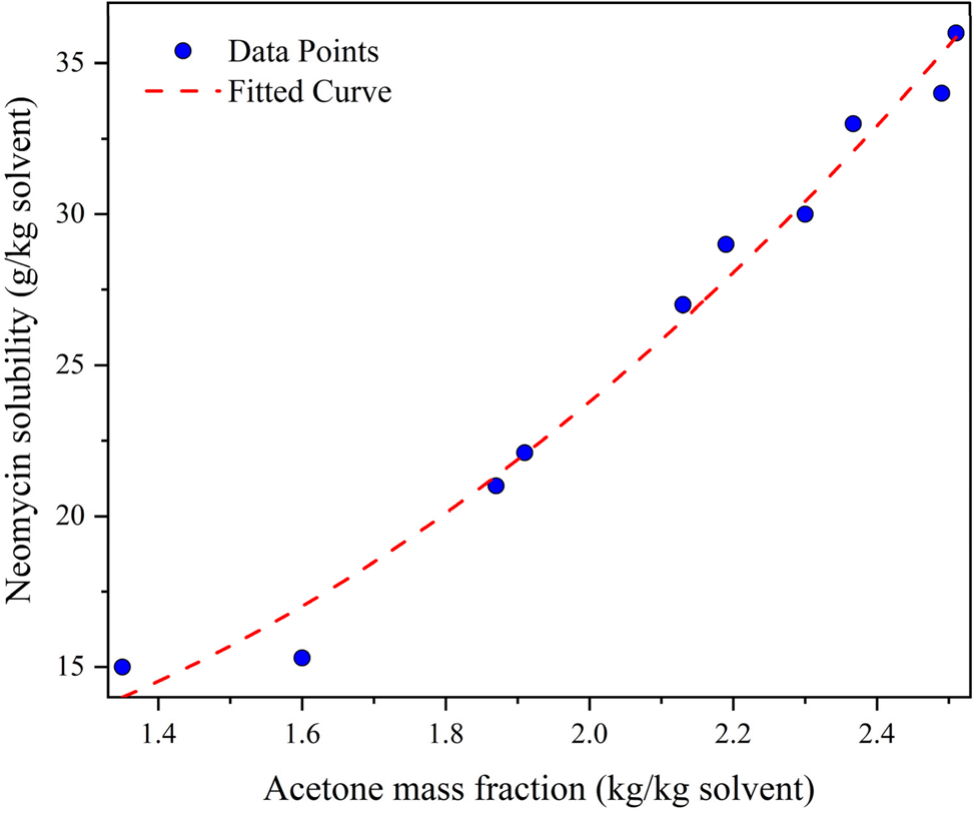
Neomycin solubility *versus* acetone mass fraction at 25 °C for a stirring speed of 300 rpm.

To measure the neomycin solubility, approximately 0.2 g of neomycin was added to 10 g of a solvent solution (which included both solvent and antisolvent). The solution was then stirred for 24 hours at 25 °C and at a mixing speed of 300 rpm to ensure that the solids settled properly. As this period elapsed, the clear phase of the solution was separated from the solid sediments and filtered through a 0.45 μm filter to remove any suspended particles. The concentration of the saturated solution in a sample vial was then calculated through gravimetric analysis (the solvent was evaporated in a vacuum oven and the dried residue was weighed by gravimetric analysis) and the pertinent results are presented in Fig. 14. These tests were performed in two series for various solvent compounds (acetone mass fractions of 1.35, 1.6, 1.87, 1.91, 2.13, 2.19, 2.3, 2.367, 2.49 and 2.51).

### 4.4. Determination of induction time, supersaturation, solubility and nucleation mechanism in the presence of PVP

In this study, the PVP stabilizer has been utilized to examine the stability of neomycin nanoparticles and their impact on the nucleation mechanism, supersaturation, induction time and solubility of neomycin. However, it should be emphasized that the assessment of the degree of supersaturation depends on the solubility of neomycin (solute). Experimental results showed that neomycin nanoparticles were more stable in the presence of PVP. Furthermore, it became apparent that the lack of accumulation and agglomeration of neomycin nanoparticles after supersaturation, as well as the stability of nanoparticles for more than a few days, was due to the presence of the PVP stabilizer.^41,70–73^Table 2 shows the induction time, solubility and supersaturation (*S*) at two initial concentrations of 0.6 and 0.8 g kg^−1^ of PVP.

**Table 2:** Solubility, induction time and supersaturation concentration at the nucleation point of neomycin sulfate in the presence of PVP solution

Initial PVP concentration (g kg^−1^ solvent)	Neomycin concentration in the crystallizer (g kg^−1^)	Solubility (g kg^−1^), *C*_solubility_	Supersaturation ratio *C*_supersaturation_/*C*_solubility_	Induction time (s)
0.6	20	15	1.33	67
	30	21	1.43	47
	40	27	1.48	39
	50	30	1.66	31
	60	34	1.76	23
0.8	20	15.3	1.31	78
	30	22.1	1.358	59
	40	29	1.38	50
	50	33	1.51	36
	60	36	1.67	27

**Fig. 15:**
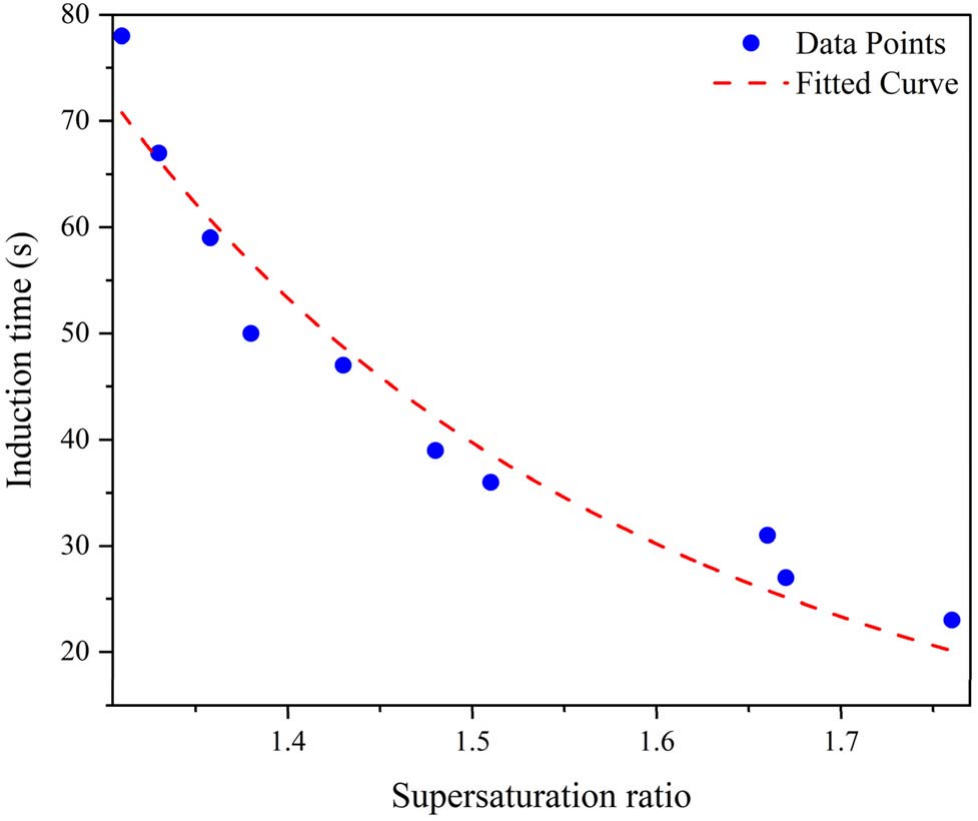
Induction time changes with supersaturation in the presence of PVP at 25 °C.

The relationship between supersaturation and induction time was investigated for different degrees of supersaturation at 25 °C and with a stirring speed of 300 rpm and the results are presented in Fig. 15. According to these results, in all experiments, higher supersaturation corresponded to less induction time, which is entirely consistent with previous studies.^74^ The reduced induction time at higher reactant concentrations can be attributed to several factors. As supersaturation increases, more molecular collisions and faster formation of critical clusters occur, causing a reduction in the size of the critical nucleus. These factors contribute to the observed decrease in induction time.^75^ With the gradual increase of supersaturation and the transition into an area with high supersaturation, the collisions of soluble molecules increase and their high concentration in the solution starts nucleation faster and reduces the induction time.^76^ These experiments were repeated twice for various solvent–antisolvent mixtures with acetone mass fractions of 1.35, 1.6, 1.87, 1.91, 2.13, 2.19, 2.3, 2.367, 2.49, and 2.51, to determine how the solvent to antisolvent ratio would impact the solubility and supersaturation.

**Fig. 16:**
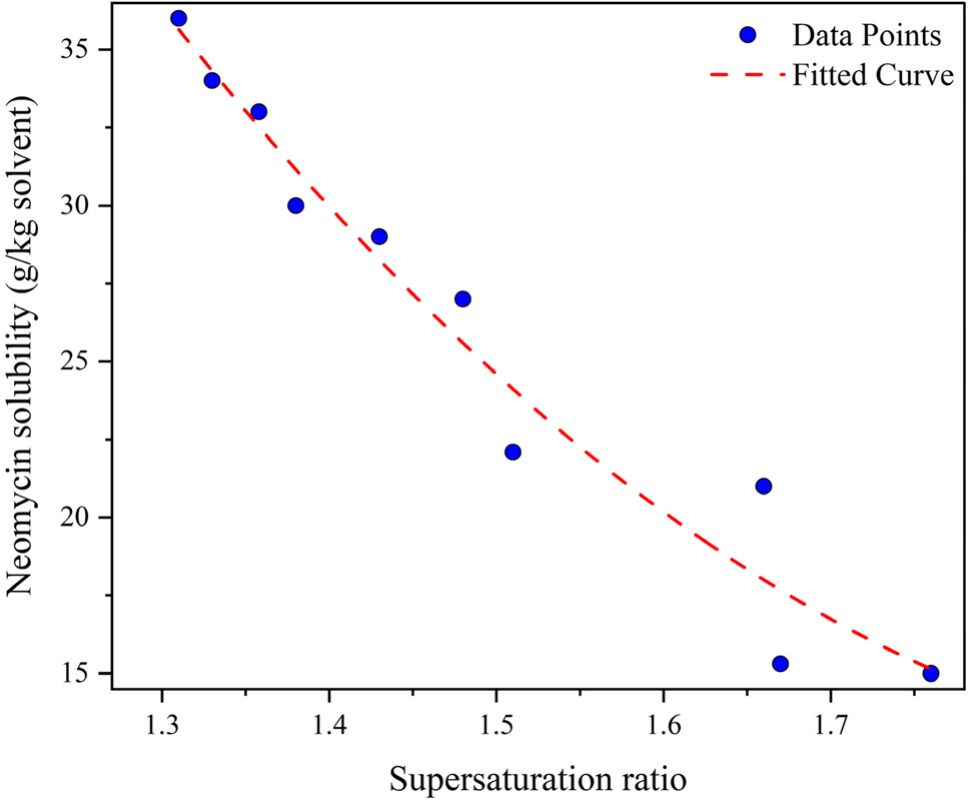
Effect of supersaturation on the neomycin solubility.

The supersaturation ratio in a system is directly affected by the solubility of the solute in the solvent and solvent–antisolvent mixture. Accurate measurement and prediction of solubility under the influence of supersaturation are essential for understanding crystal growth dynamics. In this regard, the effect of supersaturation on solubility has been investigated and shown in Fig. 16. As can be seen, the solubility decreases as supersaturation increases. The addition of antisolvent (acetone) increases supersaturation and decreases the solubility of the solute (neomycin) in solution. The combination of a solvent–antisolvent mixture (double-distilled water + acetone) that results in minimum solubility leads to maximum supersaturation.^77,78^

**Fig. 17:**
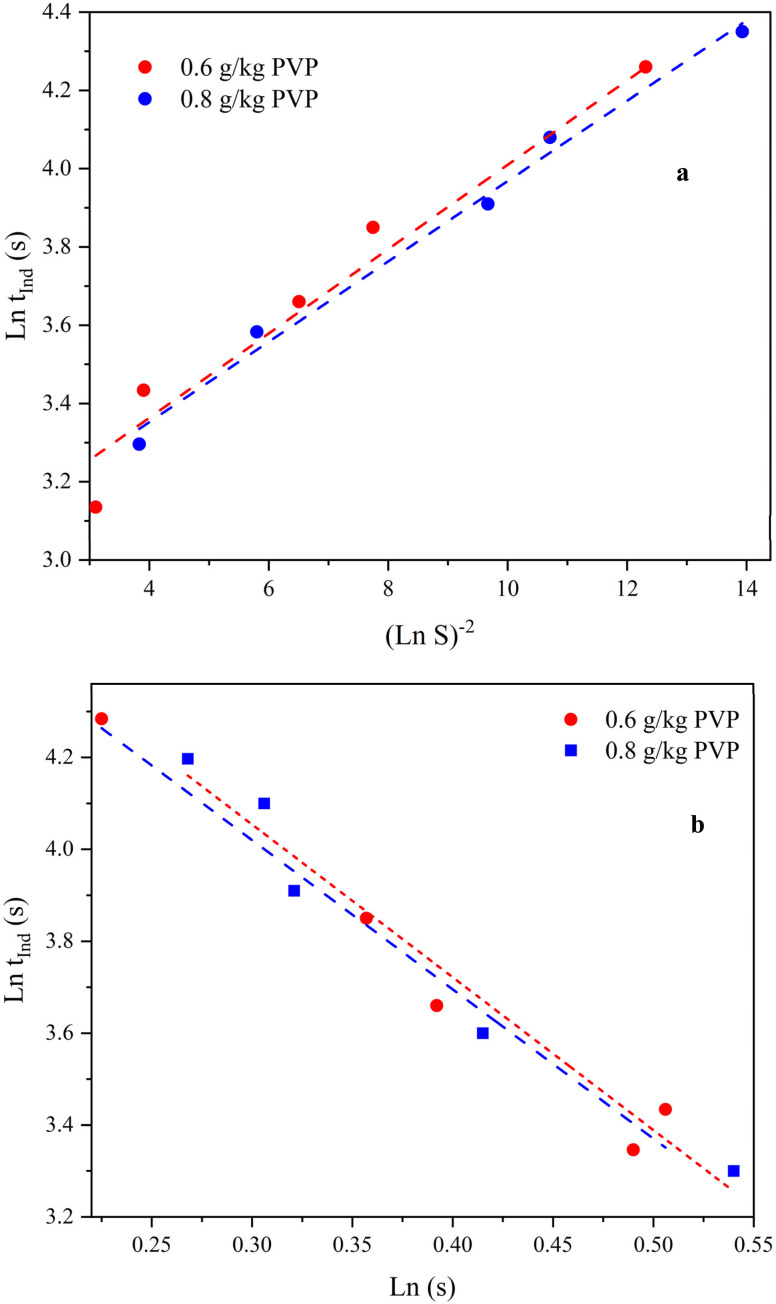
(a) ln *t*_ind_*vs.* 1/(ln *S*)^2^ and (b) ln *t*_ind_*vs.* ln *S* at a temperature of 25 °C and initial concentrations of 0.6 and 0.8 g kg^−1^ of PVP.

By plotting ln *t*_ind_*vs.* ln *S* and ln *t*_ind_*vs.* 1/(ln *S*)^2^, the nucleation mechanism can be identified. According to eqn (3), if the relationship between ln *t*_ind_ and 1/ln(*S*)^2^ is linear, then the nucleation mechanism will be of primary type. On the other hand, if the relationship between ln *t*_ind_ and ln *S* is linear, secondary nucleation is known as the dominant mechanism (according to eqn (5)). In this respect, ln *t*_ind_*vs.* 1/(ln *S*)^2^ and ln *S* at 25 °C for initial concentrations of 20, 30, 40, 50, and 60 g kg^−1^ of neomycin sulfate and initial concentrations of 0.6 and 0.8 g kg^−1^ of PVP are illustrated in Fig. 17(a and b).

Considering the linear state of both graphs, the *R*^2^ value should be checked in both graphs to understand the nucleation mechanism. In Table 3, the *R*^2^ values according to Fig. 17a and b for primary and secondary nucleation mechanisms are calculated at initial concentrations of 0.6 and 0.8 g kg^−1^ of PVP. According to the higher values of *R*^2^ for the primary nucleation mechanism, it can be concluded that the primary nucleation mechanism is the dominant nucleation mechanics.

**Table 3:** Correlation coefficients of nucleation in the presence of different PVP solutions

Initial PVP concentration (g kg^−1^)	Primary nucleation model	Secondary nucleation model
	Line equation; *R*^2^	Line equation; *R*^2^
0.6	0.14*X* + 2.7768; 0.9695	−3.23*X* + 5.94; 0.9364
0.8	0.117*X* + 2.852; 0.9836	−3.192*X* + 5.043; 0.9236

Although the high *R*^2^ values led to the conclusion that the primary nucleation is prevailing, the following experimental evidence also aligned well with the results of the regression equations. Firstly, no seeding or additive methods were used to create the desired crystals, and the product was obtained from a supersaturated solution. Secondly, as shown in Fig. 2c, increasing supersaturation and reaching the solubility limit in the MSZW chart, according to the theories of J. Mullin and A. Myerson,^33,76^ more nucleation would occur at the boundary between the metastable and unstable regions, confirming that the primary nucleation was dominant over the secondary nucleation.

### 4.5. Interfacial energy

The solid/liquid interfacial energy is often assumed to be constant (an intrinsic property of the system), but its value is difficult to measure.^79,80^ Interfacial energy is a function of the solvent compositions in the solution. Several studies have found that different solvents may contribute different interfacial energies.^81–83^ Furthermore, recent research has indicated that the interfacial energy of a solution can be adjusted by changing the mass ratio of the solvent mixture. Typically, interfacial energy can be easily reduced by using various surfactants.^84^

**Fig. 18:**
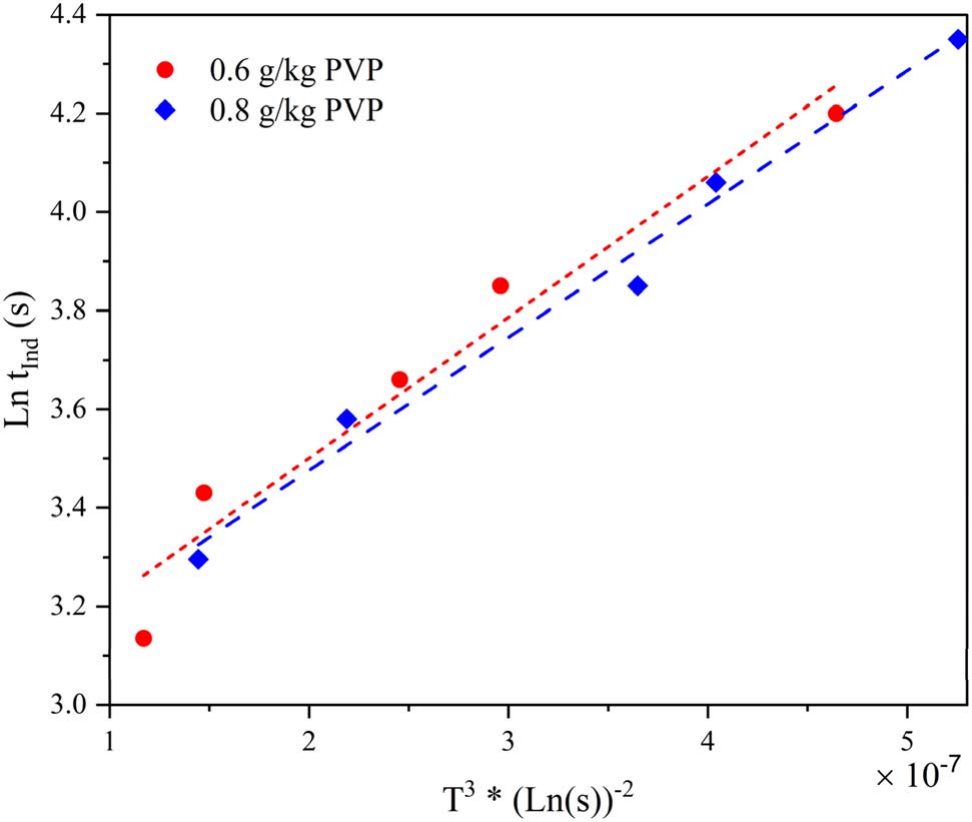
ln *t*_ind_*vs.* 1/(*T*^3^ (ln *S*)^2^) for PVP solution concentrations of 0.6 and 0.8 g kg^−1^ at 25 °C.

In order to calculate the interfacial energy, based on the theoretical framework, elaborated in section 2.2, ln *t*_ind_*versus* 1/*T*^3^(ln *S*)^2^ is plotted in Fig. 18 for two systems of containing PVP solutions with concentrations of 0.6 and 0.8 g kg^−1^ at 25 °C. The density of neomycin (*ρ* = 640 kg m^−3^) was measured with the volume difference method. Other parameters including *f* and *M* are as follows: *f* = 0.058,^85^ and *M* = 908.88 kg mol^−1^. The slope of the line (ln *t*_ind_*vs.* 1/*T*^3^(ln *S*)^2^), *R*^2^, and calculated interfacial energy for each system are shown in Table 4. It is evident that the system with an initial acetone mass fraction greater than 2.51 (kg kg^−1^ solvent) results in lower interfacial energy, indicating that increased solubility leads to decreased interfacial energy in the system. A higher concentration of PVP in the solvent mixture leads to changes in solubility, resulting in a lower surface energy of the solid–liquid interface between neomycin and the solvent mixture. This decrease in surface energy, or surface tension, is due to the presence of PVP, which alters the interfacial interactions at the solid–liquid interface, potentially affecting the crystallization process. These findings highlight the potential role of PVP as a surface active agent that can influence the crystallization behavior of neomycin and other similar compounds.^86,87^

**Table 4:** Comparison between interfacial energy for PVP solution concentrations of 0.6 and 0.8 g kg^−1^ at 25 °C

Initial PVP concentration (g kg_solvent_^−1^)	Slope (*A*)	*R* ^2^	Interfacial energy (mJ m^−2^)
0.6	2.86 × 10^5^	0.9695	7.68
0.8	2.72 × 10^5^	0.9836	8.056

### 4.6. Measurements of the metastable zone width (MSZW)

Studies have shown that the thermodynamic and kinetic properties of nucleation, including changes in solubility, supersaturation, MSZW, induction time, and nucleation rate, are profoundly affected by the presence of additives.^88^ Saturation and supersaturation are fundamental parameters for the optimization of product quality in crystallization processes. These parameters have a great influence on properties such as purity, crystal size, nucleation, crystal growth and MSZW. In other words, the maximum amount of saturation and supersaturation that a system containing solute and solvent (solvent and antisolvent) can tolerate is directly related to the determination of solubility, supersaturation and the MSZW.^89^ The MSZW of a substance depends on several factors, including the type of solvent and antisolvent, additives (stabilizers), the presence of impurities as well as crystalline grains in the solution, the stirring speed and the bulk temperature.

Supersaturation is known as the pivotal driving force in the crystallization process. The removal of crystals from a supersaturated solution is achieved through two primary mechanisms: nucleation and crystal growth. In the context of induced crystallization, the selection of a secondary solvent or antisolvent is considered crucial, as it greatly influences the desired level of supersaturation. Data related to saturation and supersaturation can be obtained by employing an antisolvent, enabling the identification of stable, metastable, and unstable regions within a ternary plot. Numerous phase diagrams, or ternary plots, have been developed to investigate solubility, molecular compounds, eutectics, and interactions among the three components, illustrating the crystallization zones for various compounds and pure substances. However, interpreting many of these ternary diagrams can be somewhat challenging concerning the systems involved in the separation region.^90^ Therefore, the regions for separation and crystal removal within the stable, metastable, and unstable zones can effectively be identified by utilizing the two-phase nucleation diagram in the crystallization process and leveraging saturation and supersaturation data.^54^

In this regard, the MSZW of neomycin as a function of weight percentage in the neomycin/water/acetone (solute/solvent/antisolvent) system in the presence of PVP has been investigated (ternary phase diagram). In the study, the stirring speed of the solution was set to be 300 rpm and the rate of addition of antisolvent was 0.05 ml min^−1^. The first set of tests was performed using a PVP solution with a concentration of 0.6 g kg^−1^. After reaching the solubility curve, with the appearance of signs of saturation and more acetone consumption, the conditions gradually moved towards low supersaturation. As a result, secondary nucleation and growth of crystals were observed in the region between the solubility curve and the supersolubility line, known as MSZW. Unlike industrial crystallization processes, the goal in this process was to achieve primary nucleation and produce smaller crystals.

In this study, supersaturation is created by adding acetone (antisolvent) to the system, which is insoluble in neomycin but miscible with water. The optimal ratio of antisolvent addition is crucial for achieving the saturated and supersaturated states.^91^ By plotting the saturation and supersaturation points using solubility and super-solubility curves, three regions were identified as outlined below:

- Stable region: no significant interaction occurred between neomycin, acetone, and water. The solubility of neomycin did not change noticeably and no crystallization occurred.

- Metastable region: thermodynamically unstable, but can be stabilized with further antisolvent addition. Spontaneous crystallization was unlikely but may occur with slight changes in conditions. Supersaturation was mild and consumed by crystal nuclei, with potential for secondary nucleation.

- Unstable region: when acetone concentration exceeded the solubility limit, the system became highly supersaturated, resulting in rapid nucleation. Accordingly, primary nucleation dominates, leading to smaller crystals while suppressing secondary nucleation and crystal growth. Crystallization also became partially spontaneous in this region.

**Fig. 19:**
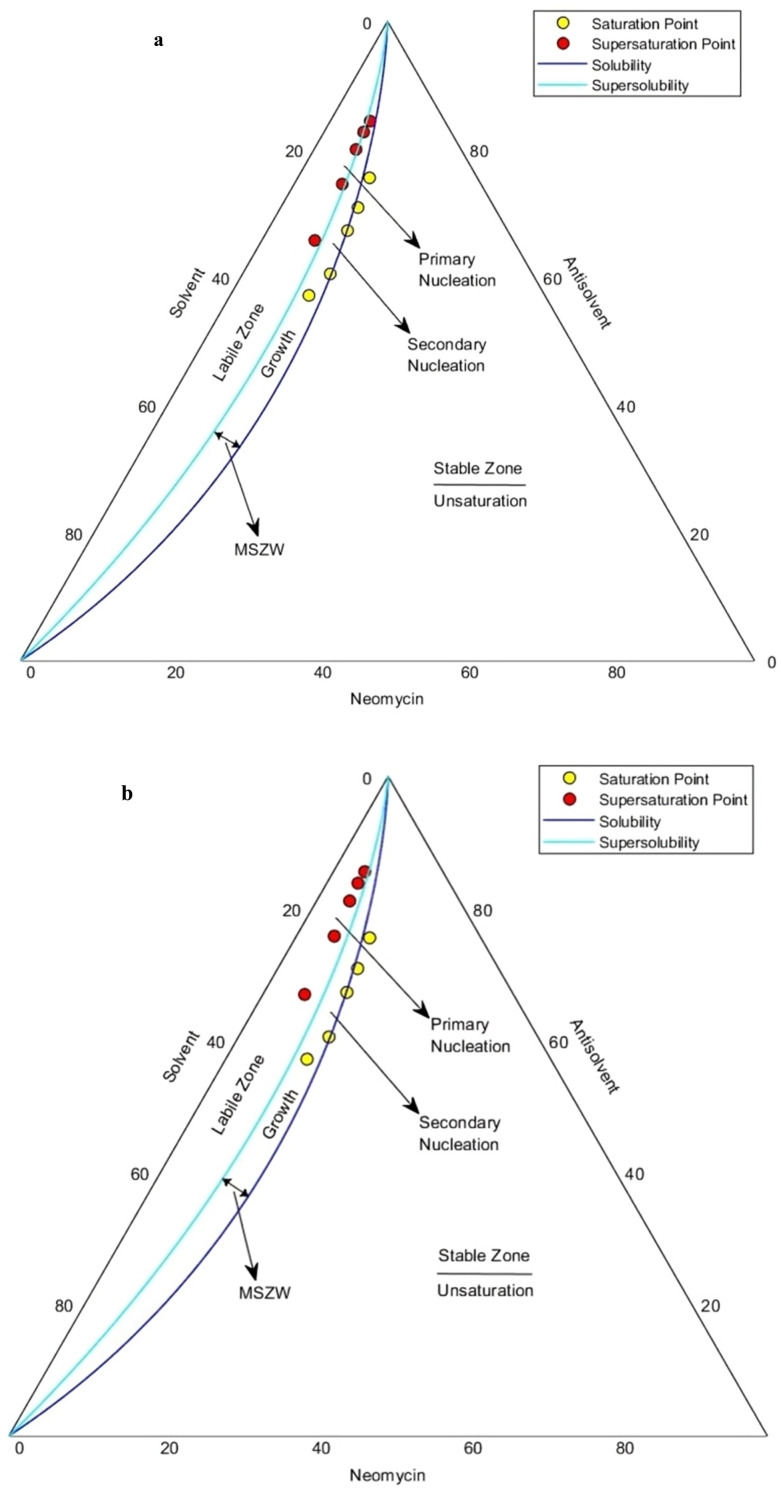
MSZW of neomycin as a function of weight percentage in the neomycin/water/acetone system in the presence of PVP solution with concentrations of (a) 0.6 g kg^−1^ and (b) 0.8 g kg^−1^.

By adding more antisolvent (acetone), the supersaturation reached higher levels near the super-solubility region, which finally led to the occurrence of homogeneous primary nucleation in the vicinity of the super-solubility curve (Fig. 19a). To investigate the effect of the stabilizer's amount, the second set of tests was conducted in the presence of PVP solution with a concentration of 0.8 g kg^−1^. As shown in Fig. 19b, secondary nucleation and growth of crystals were observed in the MSZW region as the antisolvent increased, similar to Fig. 19a. However, due to reaching higher supersaturation levels than the previous experiments, supersaturation points were formed slightly above the solubility curve. This shows that with the increase of PVP amount, heterogeneous primary nucleation occurs at higher levels of supersaturation.

In this research, a new diagram for MSZW, which is known as two phase nucleation, was designed and developed using MATLAB. This chart provides the ability to accurately estimate the amounts of neomycin, water, and acetone required for conditions of saturation, supersaturation, solubility, super-solubility, nucleation, crystal growth, as well as for determining the range of MSZW in the crystallization process. However, due to the limitations of previous research (this diagram is creatively drawn, which has not been reported before) and differences in experimental methods, it is currently not possible to compare the results of this research with other studies with respect to MSZW.

## 5. Conclusions

In this study, neomycin nanoparticles were synthesized through inductive crystallization using PVP as the stabilizer. The induction time was measured using two methods: an *in situ* turbidimeter and a reaction timer. The results indicated that the turbidimeter provided more accurate results. The prepared neomycin nanoparticles were then characterized by various analyses such as TEM, HR-TEM, SEM, FE-SEM, FT-IR, XRD, DCS, TGA, AFM, DLS, and EDX. In addition, FT-IR spectroscopy was conducted before and after their formation with PVP, confirming the presence of N–H, C–H, C=O, and O–H bonds. The nucleation mechanism was determined using classical nucleation theory, revealing that primary nucleation was the dominant process for all experiments. The impact of the solvent-to-antisolvent ratio on solubility also revealed that higher supersaturation causes a reduction in the solubility of neomycin. The supersaturation ratio was influenced by the solubility of neomycin in a mixture of water and acetone. The MSZW of neomycin in the presence of PVP has been investigated and the results showed that increased concentration of PVP caused heterogeneous primary nucleation at higher levels of supersaturation.

## Data availability

The authors confirm that all relevant data supporting the findings of this study are included within the submitted manuscript. If any additional raw data files in alternative formats are needed, they can be made available upon reasonable request from the corresponding author. The source data accompanying this paper are also provided.

## Conflicts of interest

There are no conflicts to declare.
